# Learned graphical models for probabilistic planning provide a new class of movement primitives

**DOI:** 10.3389/fncom.2012.00097

**Published:** 2013-01-02

**Authors:** Elmar A. Rückert, Gerhard Neumann, Marc Toussaint, Wolfgang Maass

**Affiliations:** ^1^Institute for Theoretical Computer Science, Graz University of TechnologyAustria; ^2^Department of Computer Science, Freie Universität BerlinGermany

**Keywords:** movement primitives, motor planning, reinforcement learning, optimal control, graphical models

## Abstract

Biological movement generation combines three interesting aspects: its modular organization in movement primitives (MPs), its characteristics of stochastic optimality under perturbations, and its efficiency in terms of learning. A common approach to motor skill learning is to endow the primitives with dynamical systems. Here, the parameters of the primitive indirectly define the shape of a reference trajectory. We propose an alternative MP representation based on probabilistic inference in learned graphical models with new and interesting properties that complies with salient features of biological movement control. Instead of endowing the primitives with dynamical systems, we propose to endow MPs with an intrinsic probabilistic planning system, integrating the power of stochastic optimal control (SOC) methods within a MP. The parameterization of the primitive is a graphical model that represents the dynamics and intrinsic cost function such that inference in this graphical model yields the control policy. We parameterize the intrinsic cost function using task-relevant features, such as the importance of passing through certain via-points. The system dynamics as well as intrinsic cost function parameters are learned in a reinforcement learning (RL) setting. We evaluate our approach on a complex 4-link balancing task. Our experiments show that our movement representation facilitates learning significantly and leads to better generalization to new task settings without re-learning.

## 1. Introduction

Efficient motor skill learning in redundant stochastic systems is of fundamental interest for both, understanding biological motor systems as well as applications in robotics.

Let us first discuss three aspects of human and animal movement generation the combination of which is the motivation for our approach: (1) its modular organization in terms of movement primitives, (2) its variability and behavior under perturbations, and (3) the efficiency in *learning* such movement strategies.

First, concerning the movement primitives (MPs) in biological motor systems, the musculoskeletal apparatus is a high-dimensional redundant stochastic system and has many more degrees-of-freedom (DoF) than needed to perform a specific action (Bernstein, 1967). A classical hypothesis is that such redundancy is resolved by a combination of only a small number of functional units, namely MPs (d'Avella et al., 2003; Bizzi et al., 2008; d'Avella and Pai, 2010). In other terms, MPs can be understood as compact parameterizations of elementary movements which allows for an efficient abstraction of the high-dimensional continuous action spaces. This abstraction has been shown to facilitate learning of complex movement skills (d'Avella et al., 2003; Schaal et al., 2003; Neumann et al., 2009).

A second important aspect about biological movement are the characteristics of motor variability under perturbations or stochasticity. If humans perform the same task several times, the resulting movement trajectories vary considerably. Stochastic optimal control (SOC), besides its high relevance in engineering problems, has proven itself as an excellent computational theory of this effect (Todorov and Jordan, 2002; Trommershauser et al., 2005). An implication of SOC, the *minimum intervention principle*, states that we should only intervene in the system if it is necessary to fulfill the given task. If the task constraints are not violated it is inefficient to suppress the inherent noise in the stochastic system. The fact that biological movements account for such principles suggests that SOC principles are involved on the lowest level of movement generation.

These biological perspectives suggest that the third aspect, efficient motor skill learning, is facilitated by this combination of MPs with low level SOC principles. While existing MP methods have demonstated efficient learning of complex movement skills (d'Avella et al., 2003; Schaal et al., 2003; Neumann et al., 2009) they lack an integration of SOC principles *within* MPs. Instead, in current approaches the parameters of the MP compactly determine the shape of the desired trajectory either directly or indirectly. This trajectory is then followed by feedback control laws. An example for an indirect trajectory parameterization are the widely used Dynamic Movement Primitives (DMPs) (Schaal et al., 2003), which use parameterized dynamical systems to determine a movement trajectory. The idea of DMPs to endowing MPs with an intrinsic dynamical system has several benefits: they provide a linear policy parameterization which can be used for imitation learning and policy search (Kober and Peters, 2011). The complexity of the trajectory can be scaled by the number of parameters (Schaal et al., 2003) and one can adapt meta-parameters of the movement such as the movement speed or the goal state of the movement (Pastor et al., 2009; Kober et al., 2010). Further, the dynamical system of a DMP is to some degree also reactive to perturbations by adapting the time progression of the canonical system depending on joint errors and thereby de- or accelerating the movement execution as needed (Ijspeert and Schaal, 2003; Schaal et al., 2003). However, the trajectory shape itself is fixed and non-reactive to the environment.

In our approach we aim to go beyond MPs that parameterize a fixed reference trajectory and instead truly integrate SOC principles within the MP. The general idea is to endow MPs with an intrinsic probabilistic planning system instead of an intrinsic dynamical system. Such a *Planning Movement Primitive* (PMP) can react to the environment by optimizing the trajectory for the specific current situation. The intrinsic probabilistic planning system is described as a graphical model that represents the SOC problem (Kappen et al. 2009; Toussaint, 2009). Training such a MP therefore amounts to learning a graphical model such that inference in the learned graphical model will generate an appropriate policy. This has several implications. First, this approach implies a different level of generalization compared to a dynamical system that generates a fixed (temporally flexible) reference trajectory. For instance, if the end effector target changes between training and testing phase, an intrinsic planning system will generalize to a new target without retraining. A system that directly encodes a trajectory would either have to be retrained or use heuristics to be adapted (Pastor et al., 2009). Second, this approach truly integrates SOC principles within the MP. The resulting policy follows the minimum intervention principle and is compliant compared to a feedback controller that aims to follow a reference trajectory.

As with DMPs, a PMP is trained in a standard RL setting. Instead of parameterizing the shape of the trajectory directly, a PMP has parameters that determine the intrinsic cost function of the intrinsic SOC system. While the reward function (typically) gives a single scalar reward for a whole movement, the learned intrinsic cost function is in the standard SOC form and defines task and control costs for every time-step of the movement. In other terms, training a PMP means to learn from a sparse reward signal an intrinsic cost function such that the SOC system will, with high probability, generate rewarded movements. Parallel to this learning of an intrinsic cost function, a PMP also exploits the data to learn an approximate model of the system dynamics. This approximate dynamics model is used by the intrinsic SOC system. Therefore, PMP learning combines model-based and model-free RL: it learns a model of the system dynamics while at the same time training PMP parameters based on the reward signal. It does not learn an approximate model of the reward function itself. We can exploit supervised learning methods such as Vijayakumar et al. (2005) and Nguyen-Tuong et al. (2008a,b) for learning the system dynamics and at the same time use policy search methods to adapt the PMP parameters that determine the intrinsic cost function. This two-fold learning strategy has the promising property of fully exploiting the data by also estimating the system dynamics instead of only adapting policy parameters.

As mentioned above, our approach is to represent the intrinsic SOC system as a graphical model, building on the recent work on Approximate Inference Control (AICO), (Toussaint, 2009). AICO generates the movement by performing inference in the graphical model that is defined by the system dynamics and the intrinsic cost function. Since we learn both from experience, all conditional probability distributions of this graphical model are learned in the RL setting. The output of the planner is a linear feedback controller for each time slice.

Our experiments show that by the use of task-relevant features, we can significantly facilitate learning and generalization of complex movement skills. Moreover, due to the intrinsic SOC planner, our MP representation implements the principles of optimal control, which allows to learn solutions of high quality which are not representable with traditional trajectory-based methods.

In the following section we review in more detail related previous work and the background on which our methods build. Section 3 then introduces the proposed PMP. In section 4 we evaluate the system on a one-dimensional via-point task and a complex dynamic humanoid balancing task and compare to DMPs. We conclude with a discussion in section 5.

## 2. Related work and background

We review here the related work based on parameterized movement policies, policy search methods and SOC.

### 2.1. Parameterized movement policies

MPs are a parametric description of elementary movements (d'Avella et al., 2003; Schaal et al., 2003; Neumann et al., 2009). We will denote the parameter vector of a MP by θ and the possibly stochastic policy of the primitive as π(**u**|**x**,*t*;θ), where **u** is the applied action and **x** denotes the state. The key idea of the term “primitive” is that several of these elementary movements can be combined not only sequentially but also simultaneously in time. However, in this paper, we want to concentrate on the parameterization of a single MP. Thus we only learn a single elementary movement. Using several MPs simultaneously is part of future work for our approach as well as for existing approaches such as Schaal et al. (2003) and Neumann et al. (2009).

Many types of MPs can be found in the literature. The currently most widely used movement representation for robot control are the DMPs (Schaal et al., 2003). DMPs evaluate parameterized dynamical systems to generate trajectories. The dynamical system is constructed such that the system is stable. In order to do so, a linear dynamical system is used which is modulated by a learnable non-linear function *f*. A great advantage of the DMP approach is that the function *f* depends linearly on the parameters θ of the MP: *f*(*s*) = Φ(*s*)^*T*^ θ, where *s* is the time or phase variable. As a result, imitation learning for DMPs is straightforward, as this can simply be done by performing a linear regression (Schaal et al., 2003). Furthermore, it also allows the use of many well-established RL methods such as policy gradient methods (Peters and Schaal 2008) or Policy Improvements by Path Integrals PI^2^ (Theodorou et al., 2010). The complexity of the trajectory can be scaled by the number of features used for modeling *f*. We can also adapt meta-parameters of the movement such as the movement speed or the goal state of the movement (Pastor et al., 2009; Kober et al., 2010). However, as the features Φ(*s*) are fixed, the ability of the approach to extract task-relevant features is limited. Yet, the change of the desired trajectory due to the change of the meta-parameters is based on heuristics and does not consider task-relevant constraints. While the dynamical system of a DMP is to some degree reactive to the environment—namely by adapting the time progression of the canonical system depending on joint errors and thereby de- or accelerating the movement execution as needed (Ijspeert and Schaal, 2003; Schaal et al., 2003)—the trajectory shape itself is fixed and non-reactive to the environment. As the DMPs are the most common movement representation, we will use it as a baseline in our experiments. A more detailed discussion of the DMP approach can be found in the Appendix.

Another type of movement representation was introduced in Neumann et al. (2009) by the movement template framework. Movement templates are temporally extended, parameterized actions, such as sigmoidal torque, velocity or joint position profiles, which can be sequenced in time. This approach uses a more complex parameterization as the DMPs. For example, it also incorporates the duration of different phases, like an acceleration or deceleration phase. The division of a movement into single phases allows the use of RL methods to learn how to sequence these primitives. However, as the approach still directly specifies the shape of the trajectory, defining complex movements for high-dimensional systems is still complicated, which has restricted the use of movement templates to rather simple applications.

An interesting movement representation arizing from analysis of biological data are muscle synergies (d'Avella et al., 2003; Bizzi et al., 2008). They have been used to provide a compact representation of electromyographic muscle activation patterns. The key idea of this approach is that muscle activation patterns are linear sums of simpler, elemental patterns, called muscle synergies. Each muscle synergy can be shifted in time and scaled with a linear factor to construct the whole activation pattern. While the synergy approach has promising properties such as the linear superposition and the ability to share synergies between tasks, except for some smaller applications (Chhabra and Jacobs, 2006), these MPs have only been used for data analysis, and not for robot control.

All the so far presented MPs are inherently local approaches. The specified trajectory and hence the resulting policy are only valid for a local (typically small) neighborhood of our initial state. If we are in a new situation, it is likely that we need to re-estimate the parameters of the MP. The generation of the reference trajectory for these approaches is often an offline process and does not incorporate knowledge of the system dynamics, proprioceptive or other sensory feedback. Because the reference trajectory itself is usually created without any knowledge of the system model, the desired trajectory might not be applicable, and thus, the real trajectory of the robot might differ considerably from the specified trajectory.

There are only few movement representations which can also be used globally, i.e., for many different initial states of the systems. One such methods is the Stable Estimator of Dynamical Systems (Khansari-Zadeh and Billard, 2011) approach. However, this method has so far only been applied to imitation learning, using the approach for learning or improving new movement skills is not straight forward. We will therefore restrict our discussion to local movement representations.

Our PMP approach is, similar as the DMPs, a local approach. In a different situation, different abstract goals and features might be necessary to achieve a given task. However, as we extract task-relevant features and use them as parameters, the same parameters can be used in different situations as long as the task-relevant features do not change. As we will show, the valid region where the local MPs can still be applied is much larger for the given control tasks in comparison to trajectory-based methods.

### 2.2. Policy search for movement primitives

Let **x** denote the state and **u** the control vector. A trajectory τ is defined as sequence of state control pairs, τ = 〈**x**_1:*T*_, **u**_1:*T*−1_〉, where *T* is the length of the trajectory. Each trajectory has associated costs *C*(τ) (denoted as extrinsic cost), which can be an arbitrary function of the trajectory. It can, but need not be composed of the sum of intermediate costs during the trajectory. For example, it could be based on the minimum distance to a given point throughout the trajectory. We want to find a MP's parameter vector θ^*^ = argmin_θ_ *J*(θ) which minimizes the expected costs J(θ)=E[C(τ)|θ]. We assume that we can evaluate the expected costs *J*(θ) for a given parameter vector θ by performing roll-outs on the real system.

In order to find θ^*^ we can apply policy search methods. Here a huge variety of possible methods exists. Policy search methods can be coarsely divided into step-based exploration and episode-based exploration approaches. Step-based exploration approaches such as Peters and Schaal 2008, Kober and Peters (2011), and Theodorou et al. (2010) apply an exploration noise to the action of the agent at each time-step of the episode. Subsequently, the policy is updated such that the (noisy) trajectories with higher reward are more likely to be repeated. In order to do this update, step-based exploration techniques strictly rely on a policy which is linear in its parameters. This is true for the DMPs (Schaal et al., 2003). Currently, the most common policy search methods are step-based approaches, including the REINFORCE (Williams, 1992), the episodic Natural Actor Critic (Peters and Schaal 2008), the PoWER (Kober and Peters, 2011), or the PI^2^ (Theodorou et al., 2010) algorithm. This also explains partially the popularity of the DMP approach for motor skill learning because DMPs are, from those introduced above, the only representation which can be used for these step-based exploration methods (apart from very simple ones like linear controllers).

However, recent research has also intensified on episode-based exploration techniques that make no assumptions on a specific form of a policy (Hansen et al., 2003; Wierstra et al., 2008; Schaal et al., 2010). These methods directly perturb the policy parameters θ and then estimate the performance of the perturbed θ parameters by performing roll-outs on the real system. During the episode no additional exploration is applied (i.e., a deterministic policy is used). The policy parameters are then updated in the estimated direction of increasing performance. Thus, these exploring methods do not depend on a specific form of parameterization of the policy. In addition, they allow the use of second order stochastic search methods that estimate correlations between policy parameters (Hansen et al., 2003; Wierstra et al., 2008 Heidrich-Meisner and Igel, 2009b). This ability to apply correlated exploration in parameter-space is often beneficial in comparison to the uncorrelated exploration techniques applied by all step-based exploration methods, as we will demonstrate in the experimental section.

### 2.3. Stochastic optimal control and probabilistic inference for planning

SOC methods such as Todorov and Li (2005), Kappen (2007), and Toussaint (2009) have been shown to be powerful methods for movement planning in high-dimensional robotic systems. The incremental Linear Quadratic Gaussian (iLQG) (Todorov and Li, 2005) algorithm is one of the most commonly used SOC algorithms. It uses Taylor expansions of the system dynamics and cost function to convert the non-linear control problem in a Linear dynamics, Quadratic costs and Gaussian noise system (LQG). The algorithm is iterative—the Taylor expansions are recalculated at the newly estimated optimal trajectory for the LQG system.

**Figure 1:**
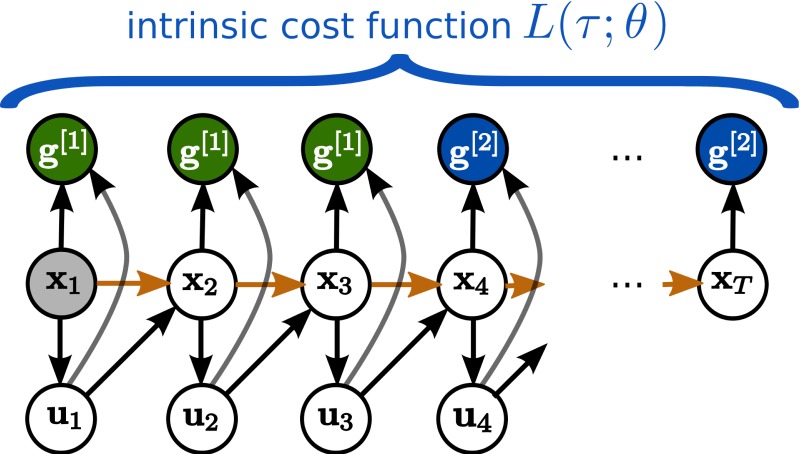
**Planning Movement Primitives are endowed with an intrinsic planning system, which performs inference in a *learned* graphical model.** States are denoted by **x**_*t*_, controls by **u**_*t*_, and the time horizon is fixed to *T* time-steps. In this example the graphical model is used to infer the movement by conditioning on two abstract goals **g**^[1]^ and **g**^[2]^, which are specified in the *learned* intrinsic cost function *L*(τ; θ).

In Toussaint (2009), the SOC problem has been reformulated as inference problem in a graphical model, resulting in the AICO algorithm. The graphical model is given by a simple dynamic Bayesian network with states **x**_*t*_, actions **u**_*t*_ and task variables **g**^[*i*]^ (representing the costs) as nodes, see Figure 1. The dynamic Bayesian network is fully specified by conditional distributions encoded by the cost function and by the state transition model. If beliefs in the graphical model are approximated as Gaussian the resulting algorithm is very similar to iLQG. Gaussian message passing iteratively re-approximates local costs and transitions as LQG around the current mode of the belief within a time slice. A difference to iLQG is that AICO uses forward messages instead of a forward roll-out to determine the point of local LQG approximation and can iterate belief re-approximation with in a time slice until convergence, which may lead to faster overall convergence. For a more detailed discussion of the AICO algorithm with Gaussian message passing see section 3.5.

Local planners have the advantage that they can be applied to high-dimensional dynamical systems, but the disadvantage of requiring a suitable initialization. Global planning (Kuffner and LaValle 2000) on the other hand does not require an initial solution, however, they have much higher computational demands. Our motivation for using only a local planner as component of a PMP is related to the learning of an intrinsic cost function.

Existing planning approaches for robotics typically use hand-crafted intrinsic cost functions and the dynamic model is either analytically given or learned from data (Mitrovic et al., 2010). PMPs use RL to train an intrinsic cost function for planning instead of trying to learn a model of the extrinsic reward directly. The reason is that a *local* planner often fails to directly solve realistically complex tasks by optimizing directly the extrinsic cost functions. From this perspective, PMPs learn to translate complex tasks to a simpler intrinsic cost function that can efficiently be optimized by a local planner. This learning is done by trial-and-error in the RL setting: the PMP essentially learns from experience which intrinsic cost function the local planner can cope with and uses it to generate good trajectories. Thereby, the RL of the intrinsic cost function can compensate the limitedness of the local planner.

## 3. Materials and methods

In this section we introduce the proposed PMPs, in particular the parameterization of the intrinsic cost function. The overall system will combine three components: (1) a regression method for learning the system dynamics, (2) a policy search method for finding the PMP parameters, and (3) a SOC planner for generating movements with the learned model and PMP parameters.

### 3.1. Problem definition

We consider an unknown dynamical system of the form

(1) $$x_{t+1}=f_{\text{Dyn}}(u_{t},x_{t})+\epsilon_{t},$$

with state variable **x**_*t*_, controls **u**_*t*_ and Gaussian noise $\epsilon_{t}\sim N(0,\sigma )$. The agent has to realize a control policy $\pi :\;x_{t}\mapsto u_{t}$, which in our case will be a linear feedback controller for each time slice. The problem is to find a policy that minimizes the expected costs of a finite-horizon episodic task. That is, we assume there exists a cost function *C*(τ), where τ=(**x**_1:*T*_, **u**_1:*T*−1_) is a roll-out of the agent controlling the system. We do not assume that the cost function *C*(τ) is analytically known or can be written as sum over individual costs for each time-step, i.e., *C*(τ) = ∑_*t*_ *h*_*t*_(**x**_*t*_,**u**_*t*_). This would imply an enormous credit assignment problem with separate costs at each time-step. Thus more generally, we only get a single scalar reward *C*(τ) for the whole trajectory. The problem is to find argmin_π_ 〈*C*(τ)〉_π_.

The system dynamics *f*_Dyn_ as well as the cost function *C*(τ) are analytically unknown. Concerning the system dynamics we can estimate an approximate model of the systems dynamics from a set of roll-outs—as standard in model-based RL. However, concerning costs, we only receive the single scalar cost *C*(τ) after a roll-out indicating the quality or success of a movement. Note that *C*(τ) is a function of the whole trajectory, not only of the final state. Learning *C* from data would be an enormous task, more complex than learning an immediate reward function $x_{t}\mapsto r_{t}$ as in standard model-based RL where **r**_*t*_ denotes the reward at time *T*.

Generally, approaches to learn *C*(τ) directly in a form useful for applying SOC methods seems an overly complex task and violates the maxim “never try to solve a problem more complex than the original.” Therefore, our approach will not try to learn *C*(τ) from data but to employ RL to learn *some* intrinsic cost function that can efficiently be optimized by SOC methods and generates control policies that, by empiricism, minimizes *C*(τ).

### 3.2. Parameterization of PMP's intrinsic cost function

In PMPs the parameters θ encode task-relevant abstract goals or features of the movement, which specify an intrinsic cost function

(2) $$L(\tau ;\theta ):=\sum_{t=0}^{T}l(x_{t},u_{t},t;\theta )+c_{p}(x_{t},u_{t}),$$

where *l* denotes the intermediate intrinsic cost function for every time-step and *c*_*p*_(**x**_*t*_, **u**_*t*_) is used to represent basic known task constraints, such as torque or joint limits. We will assume that such basic task constraints are part of our prior knowledge, thus *c*_*p*_ is given and not included in our parameterization. For the description of PMPs we will neglect the constraints *c*_*p*_ for simplicity. We will use a via-point representation for the intermediate intrinsic cost function *l*(**x**_*t*_, **u**_*t*_, *t*; θ). Therefore, parameter learning corresponds to extracting goals which are required to achieve a given task, such as passing through a via-point at a given time. As pointed out in the previous section, *L*(τ;θ) is *not* meant to approximate *C*(τ). It provides a feasible cost function that empirically generates policies that minimize *C*(τ).

There are many ways to parameterize the intermediate intrinsic cost function *l*. In this paper we choose a simple via-point approach. However, in an ongoing study we additionally implemented a desired energy state of a pendulum on a cart, which simplifies the learning problem. The movement is decomposed in *N* shorter phases with duration *d*^[*i*]^, *i* = 1,..,*N*. In each phase the cost function is assumed to be quadratic in the state and control vectors. In the *i*th phase (*t* < *d*^[1]^ for *i* = 1 and ∑^*i* − 1^_*j* = 1_ *d*^[*i*]^ < *t* ≤ ∑^*i*^_*j* = 1_ *d*^[*i*]^ for *i* > 1) we assume the intrinsic cost has the form:

(3) $$l(x_{t},u_{t},t;\theta )={\left(x_{t}-g^{\left[i\right]}\right)}^{T}R^{\left[i\right]}(x_{t}-g^{\left[i\right]})+u_{t}^{T}H^{\left[i\right]}u_{t}.$$

It is parameterized by the via-point **g**^[*i*]^ in state space; by the precision vector **r**^[*i*]^ which determines **R**^[*i*]^ = diag(exp **r**^[*i*]^) and therefore how steep the potential is along each state dimension; and by the parameters **h**^[*i*]^ which determine **H**^[*i*]^ = diag(exp**h**^[*i*]^) and therefore the control costs along each control dimension. We represent the importance factors **r**^[*i*]^ and **h**^[*i*]^ both in log space as we are only interested in the relationship of these factors. At the end of each phase (at the via-point), we multiply the quadratic state costs by the factor 1/Δ*t* where Δ*t* is the time-step used for planning. This ensures that at the end of the phase the via-point is reached, while during the phase the movement is less constraint. With this representation, the parameters θ of our PMPs are given by

(4) $$\theta =[d^{\left[1\right]},g^{\left[1\right]},r^{\left[1\right]},h^{\left[1\right]}\;\ldots \;d^{\left[N\right]},g^{\left[N\right]},r^{\left[N\right]},h^{\left[N\right]}].$$

Cost functions of this type are commonly used—and hand-crafted—in control problems. They allow to specify a via-point, but also to determine whether only certain dimensions of the state need to be controlled to the via-point, and how this trades off with control cost. Instead of hand-designing such cost functions, our method will use a policy search method to learn these parameters of the intrinsic cost function. As for the DMPs we will assume that the desired final state at time index *T* is known, and thus **g**^[*N*]^ is fixed and not included in the parameters. Furthermore, since we consider finite-horizon episodic tasks the duration of the last phase is also fixed: *d*^[*N*]^ = *T* − ∑^*N* − 1^_*i* = 1_ *d*^[*i*]^. Still, the algorithm can choose the importance factors **r**^[*N*]^ and **h**^[*N*]^ of the final phase.

### 3.3. Dynamic model learning

PMPs are endowed with an intrinsic planning system. For planning we need to learn a model of the system dynamics *f*_Dyn_ in Equation (1). The planning algorithm can not interact with the real environment, it solely has to rely on the learned model. Only after the planning algorithm is finished, the resulting policy is executed on the real system and new data points $\langle [x_{t},u_{t}],{\dot{x}}_{t}\rangle$ are collected for learning the model.

Many types of function approximators can be applied in this context (Vijayakumar et al., 2005; Nguyen-Tuong et al. (2008a,b). We use the lazy learning technique Locally Weighted Regression (LWR) (Atkeson et al., 1997) as it is a very simple and effective approach. LWR is a memory-based, non-parametric approach, which fits a local linear model to the locally-weighted set of data points. For our experiments, the size of the data set was limited to 10^5^ points implemented as a first-in-first-out queue buffer, because the computational demands of LWR drastically increase with the size of the data set. In particular we used a Gaussian kernel as distance function with the bandwidth parameters *h*_ϕ_ = 0.25 for joint angles, $h_{\dot{\phi }}=0.5$ for velocities, and *h*_*u*_ = 0 for controls^1^. For more details we refer to Chapter 4 in Atkeson et al. (1997).

### 3.4. Policy search for PMP's intrinsic cost function

Model learning takes place simultaneously to learning the parameters θ of the MP. In general this could lead to some instability. However, while the distribution *P*(**x**_*t*_) depends on the policy and the data for model learning is certainly non-stationary, the conditional distribution *P*(**x**_*t*+1_|**u**_*t*_,**x**_*t*_) is stationary. A local learning scheme as LWR behaves rather robust under such type of non-stationarity of the input distribution only. On the other hand, from the perspective of θ optimization, the resulting policies may change and lead to different payoffs *C*(τ) even for the same parameters θ due to the adaption of the learned system dynamics.

Since the resulting control policies of our PMPs depend non-linearly on the parameters θ, step-based exploration techniques can not be used in our setup. Hence, we will use the second order stochastic search method CMA (Covariance Matrix Adaptation, Hansen et al., 2003) which makes no assumptions on the parameterization of the MP.

We employ the second order stochastic search method CMA to optimize the parameters θ w.r.t. *C*(τ). The parameter space is approximated using a multivariate Gaussian distribution. Roughly, CMA is an iterative procedure that, from the current Gaussian distribution, generates a number of samples, evaluates the samples, computes second order statistics of those samples that reduced *C*(τ), and uses these to update the Gaussian search distribution. In each iteration, all parameter samples θ use the same learned dynamic model to evaluate *C*(τ). Further, CMA includes an implicit forgetting in its update of the Gaussian distribution and therefore behaves robust under the non-stationarity introduced by adaptation of the system dynamics model.

We will compare our PMP approach to both, DMPs learned with CMA policy search and DMPs learned with the state of the art step-based method PI^2^ (Theodorou et al., 2010). However, we focus in this work on the characteristics of the movement representation and place less emphasis on a specific policy search method.

Note that even if the learned model is only a rough approximation of the true dynamics, the policy search for parameters of the intrinsic cost function can compensate for an imprecise dynamics model: the policy search approach finds parameters θ of the intrinsic cost function such that—even with a mediocre model—the resulting controller will lead to low extrinsic costs in the real system.

### 3.5. Probabilistic planning algorithm

We use the probabilistic planning method AICO (Toussaint, 2009) as intrinsic planning algorithm. It offers the interpretation that a MP can be represented as graphical model and the movement itself is generated by inference in this graphical model.

The graphical model is fully determined by the learned system dynamics and the learned intrinsic cost function, see Figure 1. In order to transform the minimization of *L*(τ;θ) into an inference problem, for each time-step an individual binary random variable *z*_*t*_ is introduced. This random variable indicates a reward event. Its probability is given by

$$P(z_{t}=1|x_{t},u_{t},t)\propto \exp(-l(x_{t},u_{t},t;\theta )),$$

where *l*(**x**_*t*_, **u**_*t*_, *t*; θ) denotes the cost function for time-step *t* defined in Equation (3). AICO now assumes that a reward event *z*_*t*_ = 1 is observed at every time-step. Given that evidence, AICO calculates the posterior distribution *P*(**x**_1:*T*_, **u**_1:*T*−1_|*z*_1:*T*_ = 1) over trajectories.

We will use the simplest version of AICO (Toussaint, 2009), where an extended Kalman smoothing approach is used to estimate the posterior distribution *P*(**x**_1:*T*_, **u**_1:*T*−1_|*z*_1:*T*_ = 1). The extended Kalman smoothing approach uses Taylor expansions to linearize the system and subsequently uses Gaussian messages for belief propagation in a graphical model. Gaussian message passing iteratively re-approximates local costs and transitions as a LQG around the current mode of the belief within a time slice. For more details we refer to Toussaint (2009).

AICO is only a local optimization method and we have to provide an initial solution which is used for the first linearization. We will use the direct path (or the straight line) to the via-points **g**^[*i*]^ in Equation (3) as initial solution. Before learning the via-points **g**^[*i*]^ with *i* = 1..*N* − 1 are set to the initial state **x**_1_. The final via-point is fixed and set to the desired final state **g**^[*N*]^ = **x**_*T*_.

AICO provides us with a linear feedback controller for each time slice of the form

(5) $$u_{t}=O_{t}x_{t}+o_{t},$$

where **O**_*t*_ is the inferred feedback control gain matrix and **O**_*t*_ denotes the linear feedback controller term. This feedback control law is used as policy of the MP and is evaluated on a simulated or a real robot.

The original formulation of the AICO method (Toussaint, 2009) does not consider torque limits, which are important for many robotic experiments as well for the dynamic balancing experiments we consider in this paper. Therefore we extended the algorithm. This extension yields not only a modified form of the immediate cost function but also results in different update equations for the messages and finally different equations of the optimal feedback controller. A complete derivation of the extension including the resulting messages and the corresponding feedback controller is given in Rückert and Neumann (2012). Also the algorithm is listed in that work.

**Figure 2:**
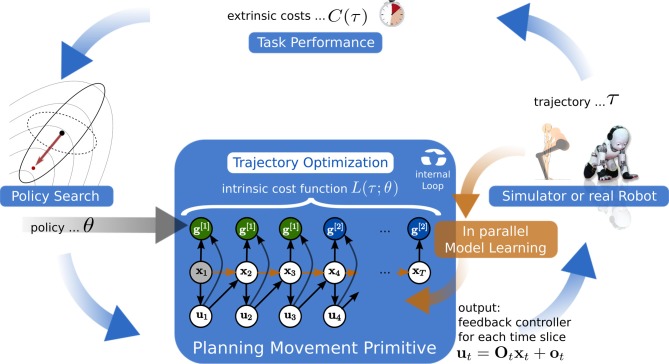
**We decompose motor skill learning into two different learning problems.** At the highest level we learn parameters θ of an intrinsic cost function *L*(τ;θ) using policy search (model-free RL). Given parameters θ the probabilistic planner at the lower level uses the intrinsic cost function *L*(τ;θ) and the learned dynamics model to estimate a linear feedback controller for each time-step (model-based RL). The feedback controller is subsequently executed on the simulated or the real robot and the extrinsic cost *C*(τ) is evaluated. Based on this evidence the policy search method computes a new parameter vector. Simultaneously we collect samples of the system dynamics $\langle [x_{t},u_{t}],{\dot{x}}_{t}\rangle$ while executing the movement primitive. These samples are used to improve our learned dynamics model which is used for planning.

On overview of the interactions between policy search of the PMP's intrinsic cost function and the planning process using AICO is sketched in Figure 2. The learning framework is organized the following: given the parameters θ from the policy search method CMA, AICO is initialized with an initial solution which is the direct path to the via-points. AICO is then used to optimize the parameterized intrinsic cost function *L*(τ;θ) to estimate a linear feedback controller for each time-step, see Equation (5). The feedback controller is subsequently executed on the simulated or the real robot and the extrinsic cost *C*(τ) is evaluated. Based on this evidence CMA will update its distribution over the policy search space and computes a new parameter vector. Simultaneously we collect samples of the system dynamics $\langle [x_{t},u_{t}],{\dot{x}}_{t}\rangle$ while executing the MP. These samples are used to improve our learned dynamics model, which is used for planning.

## 4. Results

We start our evaluation of the proposed PMP approach on a one-dimensional via-point task to illustrate basic characteristics. In order to demonstrate our approach on a more challenging dynamic robot task we choose a complex 4-link humanoid balancing task. At the end of this section we discuss an important issue: the computational time of PMPs for simulated and real world tasks.

In our experiments, we focus on investigating the optimality of the solution, the robustness to noise for learning, and the generalizability to different initial or final states. For the 4-link task we additionally demonstrate how model learning influences the learning performance.

For a comparison we take the commonly used DMPs as a baseline where we use the newest version of the DMPs (Pastor et al., 2009) as discussed in detail in Appendix A. As described above we use 2nd order stochastic search to learn the PMP and DMP parameters. In order to compare to a more commonly used policy search algorithm we additionally test the PI^2^ algorithm (Theodorou et al., 2010) for learning the DMP parameters. For all experiments we empirically evaluate the optimal settings of the algorithms (such as the exploration rate of CMA and PI^2^, the number of centers for the DMPs, or the number of via-points for the PMPs), which are listed in Appendix B.

### 4.1. One-dimensional via-point task

**Figure 3:**
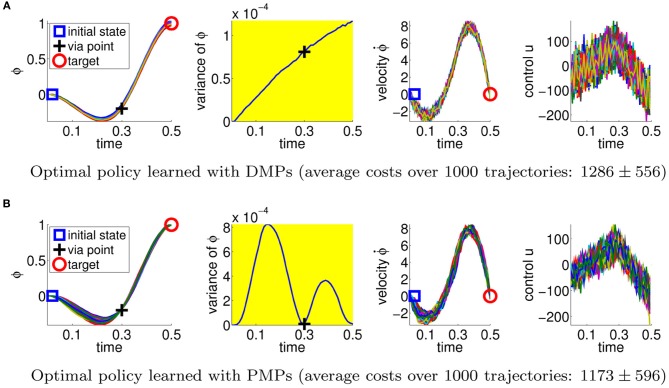
**This figure illustrates the best available policies for the DMPs and the PMPs for the via-point task.** From left-to-right shown are the point mass trajectories, the variance of these trajectories, the velocity of the point mass, and the applied accelerations. The agent has to pass the via-point at *t*_*v*_ = 0.3 s and deal with the stochasticity of the system (Gaussian control noise with a variance of 20^2^). The plots show 100 trajectories reproduced with the optimal parameters for the DMPs **(A)** and 100 trajectories with the (hand-crafted) optimal parameters for PMPs **(B)**. The PMP approach is able to reduce the variance of the movement if it is relevant for the task, while the DMPs can only suppress the noise in the system throughout the trajectory in order to get an acceptable score. This advantage is also reflected by the average costs over 1000 trajectories. The DMP solution achieved cost values of 1286±556 whereas the PMP result was 1173±596.

In this task the agent has to control a one-dimensional point mass of 1 kg. The state at time *T* is denoted by $x_{t}={\left[\phi_{t},{\dot{\phi }}_{t}\right]}^{T}$ and we directly control the acceleration *u*_*t*_. The time horizon was limited to *T* = 50 time-steps, which corresponds to a simulation time of 0.5 s with a time-step of Δ*t* = 10 ms. Starting at **x**_1_ = [0, 0]^*T*^ the agent has to pass through a given via-point *g*_*v*_ = −0.2 at *t*_*v*_ = 30. The velocity of the point mass at the via-point is not specified and can have any value. The final target *g*_*T*_ was set to 1. The movement is shown in Figure 3. For this task we define the extrinsic cost function:

$$C(\tau )=10^{4}({\dot{\phi }}_{T}{}^{2}+10{\left(g_{T}-\phi_{T}\right)}^{2})+10^{5}{\left(g_{v}-\phi_{t_{30}}\right)}^{2}+5\cdot 10^{-3}\sum_{t=1}^{T}u_{t}^{2}.$$

The first two terms punish deviations from the target *g*_*T*_ and the via-point *g*_*v*_, where ϕ_*t*_30__ denotes the first dimension of the state $x_{t}={\left[\phi_{t},{\dot{\phi }}_{t}\right]}^{T}$ at time index 30. The target should be reached with zero velocity at *T* = 50. The last term punishes high energy consumption where *u*_*t*_ denotes the applied acceleration. The control action is noisy, we always add a Gaussian noise term with a standard deviation of σ = 20 to the control action. As this is a very simple task, we use it just to show different characteristics of the DMPs (using 10 Gaussians for that representation was optimal) and PMPs (apparently 2 via-points are sufficient for this task).

A quite similar task has been used in Todorov and Jordan (2002) to study human movement control. The experiments showed that humans were able to reach the given via-points with high accuracy, however, in between the via-points, the trial-to-trial variability was rather high. This is a well-known concept from optimal control, called the *minimum intervention principle*, showing also that human movement control follows basic rules of optimal control.

#### 4.1.1. Optimality of the solutions

We first estimate the quality of the *best available* policy with the DMP and the PMP approach. We therefore use the PMPs with two via-points and set the parameters θ per hand. As we are using a linear system model and a simple extrinsic cost function, the PMP parameters can be directly obtained by looking at the extrinsic costs. As the PMPs use the AICO algorithm, which always produces optimal policies for LQG systems, the PMP solution is the optimal solution. We subsequently use the mean trajectory returned by AICO and use imitation learning to fit the DMP parameters. We also optimized the feedback controllers used for the DMPs^2^). In Figure 3 we plotted 100 roll-outs of the DMP and PMP approach using this optimal policies. The second column illustrates the trial-to-trial variability of the trajectories. The optimal solution has minimum variance at the via-point and the target. As expected this solution is reproduced with the PMP approach, because the parameters of the PMPs are able to reflect the importance of passing through the via-point. The DMPs could not adapt the variance during the movement because the used (optimized) feedback controller uses constant controller gains. As we can see, the variance of the DMP trajectory is simply increasing with time.

Comparing the optimal solutions we find that PMPs, in contrast to DMPs, can naturally deal with the inherent noise in the system. This is also reflected by the average cost values over 1000 trajectories, 1286±556 for the DMPs and 1173±596 for the PMPs. The ± symbol always denotes the standard deviation. PMPs perform significantly better than DMPs (*t*-test: *p* < 10^−3^).

#### 4.1.2. Robustness to noise for learning

**Figure 4:**
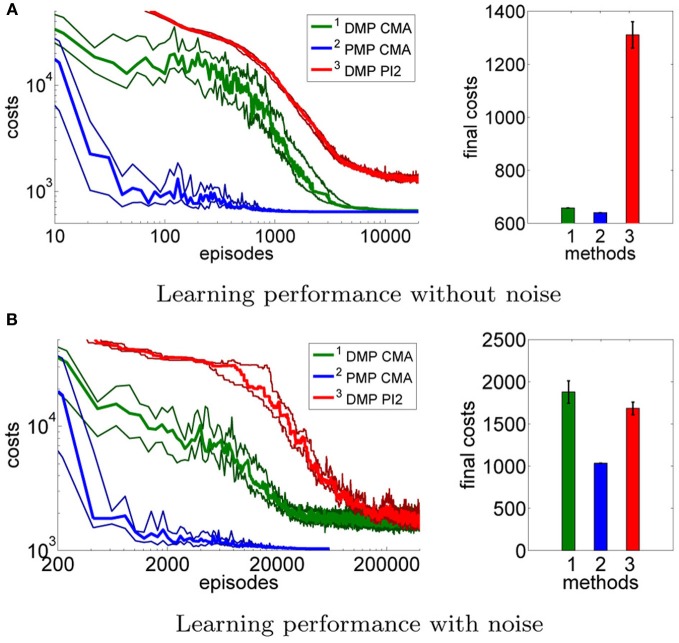
**This figure illustrates the learning performance of the two movement representations, DMPs and PMPs, for the one-dimensional via-point task.** Illustrated are mean values and standard deviations over 15 runs after CMA policy search. In addition, we also compare to the PI^2^ approach (Theodorou et al., 2010) which we could only evaluate for the DMP approach. Without noise the final costs of the two representations are similar if CMA policy search is used **(A)**. In the second example **(B)** we use zero-mean Gaussian noise with σ = 20 for the controls. In this setup we needed to average each performance evaluation for CMA over 20 roll-outs. For both setups the PMPs could considerably outperform the DMPs in terms of learning speed. For the noisy setup the PMPs could additionally produce policies of much higher quality as they can adapt the variance of the trajectories to the task constraints. PI^2^ could not find as good solutions as the CMA policy search approach in both setups.

This advantage would not be very useful if we were not able to learn the optimal PMP parameters from experience. Next we test using CMA policy search to learn the parameters for the DMPs and the PMPs. In addition, in order to compare to a more commonly used policy search method, we also compare to the PI^2^ approach (Theodorou et al., 2010) which we could only evaluate for the DMP approach. We evaluated the learning performance in the case of no control noise, Figure 4A, and in the case of control noise σ = 20, Figure 4B performing 15 runs.

Without control noise the quality of the learned policy found by 2nd order search is similar for the DMPs (657.5±0.18) and the PMPs (639.6±0.01). PI^2^ could not find as good solutions as the stochastic search approach. The reason for this is that PI^2^ could not find the very large weight values which are needed for the last few centers of the DMPs in order to have exactly zero velocity at the final state (note that the weights of the DMPs are multiplied by the phase variable *s* which almost vanishes in the end of the movement and therefore these weight values have to be very high). Because CMA policy search uses second order information, such large parameter values are easily found. This comparison clearly shows that using 2nd order search for policy search is justified. If we compare the learning speed in terms of required episodes or roll-outs between DMPs and PMPs, we find an advantage for PMPs which could be learned an order of magnitude faster than the DMPs.

The second experiment (with control noise of σ = 20) was considerably harder to learn. Here, we needed to average each performance evaluation over 20 roll-outs. The use of more sophisticated extensions of CMA (Heidrich-Meisner and Igel, 2009a) which can deal with noisy performance evaluations and hence improve the learning speed of CMA policy search in the noisy setup is part of future work. In Figure 4B we find that the PMPs could be learned an order of magnitude faster than the DMPs. As expected from the earlier experiment, the PMPs could find clearly better solutions as the DMPs as they can adapt the variance of the trajectory to the task constraints. Again, PI^2^ showed a worse performance than 2nd order search. Illustrated are mean values and standard deviations over 15 runs of learning (1034±1.46 for the PMPs and 1876±131 for the DMPs using CMA). To compare these results to the optimal costs we evaluated the best learned policies of both approaches and generated 1000 trajectories. The learned solution for the PMPs was similar to the hand-coded optimal solution, 1190±584 versus costs of 1173±596 for the optimal solution. DMPs achieved costs of 1478±837, illustrating that, eventhough the DMPs are able to represent much better solutions with costs of 1286±556 (see Figure 3), it is very hard to find this solution.

In Table 1, we show the mean and variance of the found parameters averaged over 15 runs for the first via-point in comparison to the optimal PMP parameters. We can see that the found parameters matched the optimal ones. Interestingly, in the experiment with no noise, the found parameters had a larger deviation from the optimal ones, especially for the first via-point *g*^[1]^ in Table 1. The reason for this is the simple observation that without noise, we can choose many via-points which results in the same trajectory, whereas with noise we have to choose the correct via-point in order to reduce the variance of the trajectory at this point in time.

**Table 1:** **Learned parameters using PMPs for the via-point task (1st via-point)**.

**Scenario**	***d*^[1]^**	***g*^[1]^**	**log(r^[1]^)**	**log(h^[1]^)**
Optimal	0.3	**−0.2**	[5, 0]	−2.3
No noise	0.29 ± 0.01	**−0.27** ± 0.03	[4.08 ± 4.18, −0.8 ± −0.77]	−3.05 ± −4
With noise	0.29 ± 0.01	**−0.23** ± 0.05	[4.93 ± 5.29, −0.31 ± −0.12]	−2.85 ± −3

The symbol ± denotes the standard deviation.

#### 4.1.3. Generalizability to different task settings

Next, we investigate the ability of both approaches to adapt to different situations or to adapt to different priors. In the previous task, Figure 3 the initial and the target state were assumed as prior knowledge. The movement was learned for the initial state ϕ_1_ = 0 and for the target state ϕ_*T*_ = 1. We want to investigate if the same learned parameters can be re-used to generate different movements, e.g., used for different initial or target states.

For PMPs we use the new initial or final states denoted by **x**_1_ and **g**^[*N*]^ in the graphical model in Figure 1 and re-plan the movement (using the same learned parameters). The change of the initial state or the target state is also allowed by the DMP framework. However, how the movement is generalized to these new situations is based on heuristics (Pastor et al., 2009) and does not consider any task constraints (in this example to pass through the via-point).

**Figure 5:**
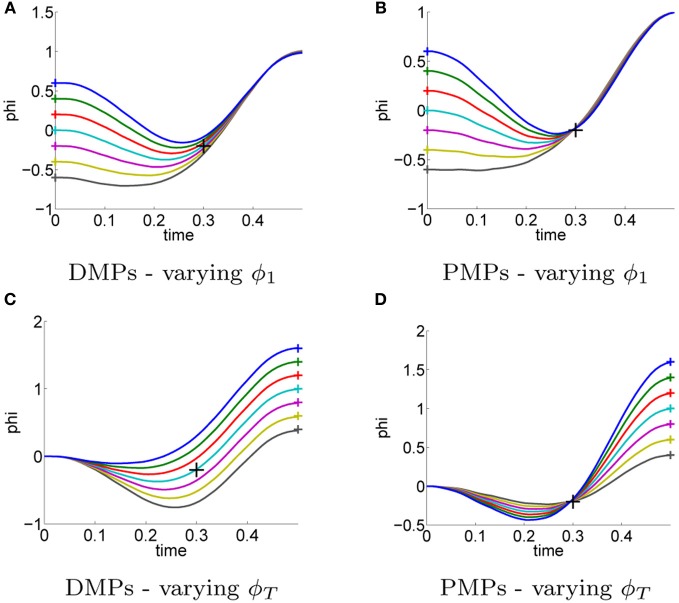
**For the previous task illustrated in Figure 3 the movement was learned for a single initial state ϕ_1_ = 0 and a single target state ϕ_*T*_ = 1.** The initial and the target state were assumed as prior knowledge. In this experiment we evaluated the generalization of the learned policies to different initial states ϕ_1_ ∈ {−0.6, −0.4, −0.2, 0, 0.2, 0.4, 0.6} **(A–B)** and different target states ϕ_*T*_ ∈ {1.5, 1.25, 1, 0.75, 0.5} **(C–D)**. Always the same parameters θ have been used, i.e., the parameters were not re-learned. Illustrated are the mean trajectories. The DMPs **(A,C)** are not aware of task-relevant features and hence do not pass through the via-point any more. **(B,D)** PMPs can adapt to varying initial or target states with small effects on passing through the learned via-point.

In Figure 5 the learned policies are applied to reach different initial states ϕ_1_ ∈ {−0.6, −0.4, −0.2, 0, 0.2, 0.4, 0.6} (Figures 5A,B) and different goal states ϕ_*T*_ ∈ {1.5, 1.25, 1, 0.75, 0.5} (Figures 5C,D). All plots show the mean trajectory. In order to change the initial or the target state of the movement we have to change the point attractor of the DMPs, which changes the complete trajectory. Due to this heuristic, the resulting DMP trajectories shown in Figures 5A,C do not pass through the via-point any more. Note that we use a modified version of the DMPs (Pastor et al., 2009) which has already been built for generalization to different initial or target points. The PMPs on the other hand still navigate through the learned via-point when changing the initial or the goal state as shown in Figures 5B,D.

#### 4.1.4. Concluding summary

As we have seen the PMPs implement all principles of optimal control, which allows to learn solutions for stochastic systems of a quality which is not representable with traditional trajectory-based methods such as the DMPs. The optimal movement trajectory could be learned from scratch up to one order of magnitude faster compared to DMPs. This difference was even more visible in the stochastic case, where the DMPs needed more than 30,000 episodes to find satisfactory solutions. In the setting with control noise the learned parameters matches the optimal ones because only by the use of noise the parameters are uniquely determined. Finally the PMPs could extract the task-relevant feature, the via-point. Even if the task changes—e.g., the initial or the final state are changed, the movement trajectory still passes through the learned via-point. The DMPs on the other hand heuristically scale the trajectory which offers no control for fulfilling task-relevant constraints.

With DMPs 12 parameters were learned, where we used 10 Gaussian kernels and optimized 2 control gains. For the PMPs 2 via-points were sufficient, where the last one was fixed. However, for both via-points we could specify 3 importance weights. Thus, in total 8 = 2 + 3 + 3 parameters were learned.

### 4.2 Dynamic humanoid balancing task

**Figure 6:**
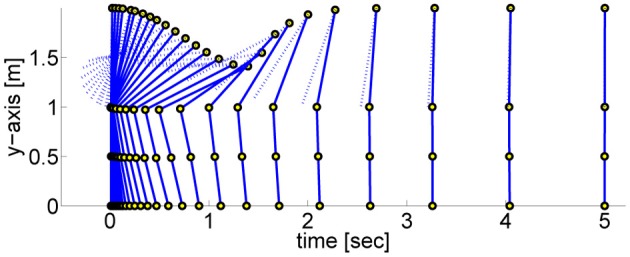
**This figure illustrates a dynamic balancing movement learned using the proposed Planning Movement Primitives.** The 4-link robot modeling a humanoid (70 kg, 2 m) gets pushed from behind with a specific force (*F* = 25 Ns) and has to move such that it maintains balance. The optimal policy is to perform a fast bending movement and subsequently return to the upright robot posture. The circles denote the ankle, the knee, the hip, and the shoulder joint. The arm link is drawn as dotted line to differentiate it from the rest of the body.

In order to assess the PMPs on a more complex task, we evaluate the PMP and DMP approach^3^ on a dynamic non-linear balancing task (Atkeson and Stephens, 2007). The robot gets pushed with a specific force *f* and has to keep balance. The push results in an immediate change of the joint velocities. The motor torques are limited, which makes direct counter-balancing of the force unfeasible. The optimal strategy is therefore to perform a fast bending movement and subsequently return to the upright position, see Figure 6. This is a very non-linear control problem, applying any type of (linear) balancing control or local optimal control algorithm such as AICO with the extrinsic cost function fails. Thus, we have to use a parametric movement representation. Like in the previous experiment, we take the DMP (Schaal et al., 2003) approach as a baseline.

We use a 4-link robot as a simplistic model of a humanoid (70 kg, 2 m) (Atkeson and Stephens, 2007). The eight-dimensional state **x**_*t*_ is composed of the arm, the hip, the knee and the ankle positions and their velocities. Table 1 in Rückert and Neumann (2012) shows the initial velocities (resulting from the force *f* which always acts at the shoulder of the robot) and the valid joint angle range for the task. In all experiments the applied force was *F* = 25Ns. If one of the joints leaves the valid range the robot is considered to be fallen. Additionally to the joint limits, the controls are limited to the intervals [±250, ±500, ±500, ±70] Nm (arm, hip, knee, and ankle). For more details we refer to Atkeson and Stephens (2007).

Let *t*_*s*_ be the last time index where the robot has not fallen and let **x**_*t*_*s*__ be the last valid state. The final state or resting state (upright position with zero velocity) is denoted by **x**_*r*_. The movement was simulated for 5 s with a Δ*t* = 10 ms resulting in *T* = 500 time-steps. As extrinsic cost function *C*(τ) we use:

(6) $$C(\tau )=20{\left(t_{s}-T\right)}^{2}+{\left(x_{t_{s}}-x_{r}\right)}^{T}R_{E}(x_{t_{s}}-x_{r})+\sum_{t=1}^{t_{s}}u_{t}^{T}H_{E}u_{t}.$$

The first term (*t*_*s*_ − *T*)^2^ is a punishment term for falling over. If the robot falls over, this term typically dominates. The precision matrix **R**_*E*_ determines how costly it is not to reach **x**_*r*_. The diagonal elements of **R**_*E*_ are set to 10^3^ for joint angles and to 10 for joint velocities. Controls are punished by **H**_*E*_ = 5 · 10^−6^ **I**. Because of the term (*t*_*s*_ − *T*)^2^ we cannot directly encode the extrinsic cost function as a sum of intermediate costs, which is usually required for SOC algorithms. But we can use PMPs to transform this reward signal into an intrinsic cost function for a local probabilistic planner.

#### 4.2.1. Optimality of the solutions

**Figure 7:**
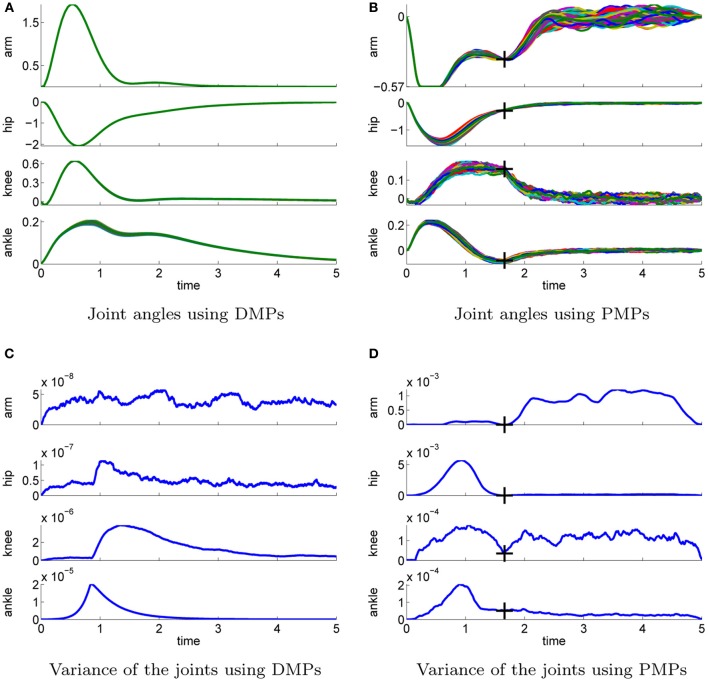
**This figure illustrates the best learned policies for the 4-link balancing task using DMPs (left column) and PMPs (right column).** Shown are the joint angle trajectories **(A–B)** and the variance of these trajectories **(C–D)**. The applied controls are illustrated in Figure 8. We evaluated 100 roll-outs using the same parameter setting θ for each approach. The controls were perturbed by zero-mean Gaussian noise with σ = 10 Ns. PMPs can exploit the power of stochastic optimal control and the system is only controlled if necessary, see the arm joint trajectories in **(B)**. The learned via-points are marked by crosses in **(B)** and **(C)**. For DMPs the variance of the joint trajectories **(C)** is determined by the learned controller gains of the inverse dynamics controller. As constant controller gains are used the variance can not be adapted during the movement and is smaller compared to **(D)**. For DMPs the best available policy achieved cost values of 568 whereas the best result using PMPs was 307.

**Figure 8:**
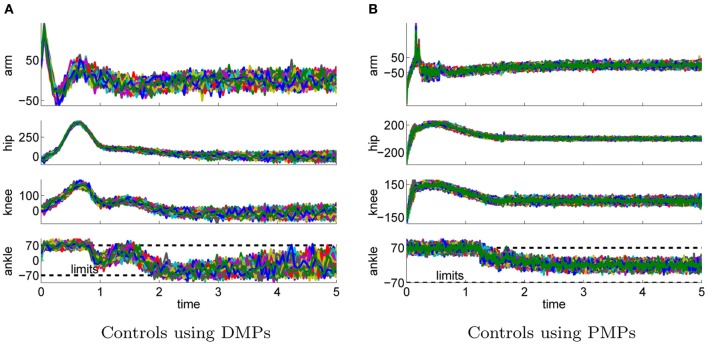
**This figure illustrates the controls of best learned policies for the 4-link balancing task using DMPs (A) and PMPs (B).** The corresponding joint angle trajectories are shown in Figure 7. The controls were perturbed by zero-mean Gaussian noise with σ = 10 Nm. Shown are 100 roll-outs using the same parameter setting θ for each approach. The dotted horizontal lines in the last row indicate the control constraints.

We use additive zero-mean Gaussian noise with a standard deviation σ = 10. In contrast to the simple via-point task where imitation learning was used to compare the trajectories shown in Figure 3 are the policies for the 4-link task learned from scratch. Figure 7 illustrates the best learned policies for DMPs (left column) and PMPs (right column). Shown are the joint angle trajectories (Figures 7A,B) and the variance of these trajectories (Figures 7C,D). The corresponding controls are illustrated in Figure 8. We evaluated 100 roll-outs of the best policies found by both approaches. While the DMPs cannot adapt the variance during the movement Figure 7C, the PMPs can exploit the power of SOC and are able to reduce the variance at the learned via-point (marked by crosses) Figure 7D. As the PMPs are able to control the variance of the trajectory, we can see that the variance of the movement is much higher compared to the DMPs (Figures 7C,D). Accuracy only matters at the via-points. We can also see that the arm trajectory has a high variance after the robot is close to a stable up-right posture Figure 7B, because it is not necessary to strictly control the arm in this phase. The best found policy of the DMPs had costs of 568 while the best result using PMPs was 307. This strongly suggests that it is advantageous to reduce the variance at certain points in time in order to improve the quality of the policy.

#### 4.2.2. Robustness to noise for learning

**Figure 9:**
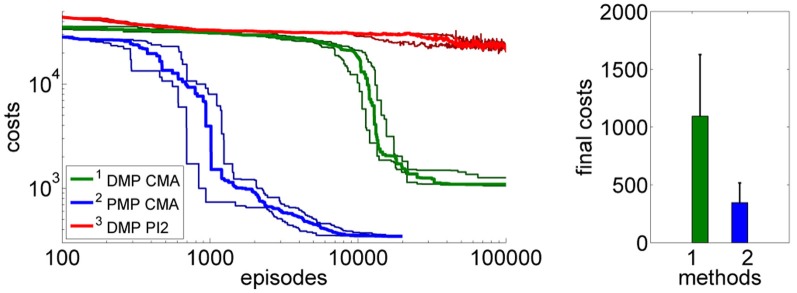
**The figure illustrates the learning performance of the two movement representations, DMPs and PMPs for the 4-link balancing task.** Illustrated are mean values and standard deviations over 20 runs after CMA policy search. The controls (torques) are perturbed by zero-mean Gaussian noise with σ = 10 Nm. The PMPs are able to extract characteristic features of this task which is a specific posture during the bending movement, shown in Figure 7B. Using the proposed Planning Movement Primitives good policies could be found at least one order of magnitude faster compared to the trajectory-based DMP approach. Also, the quality of the best-found policy was considerably better for the PMP approach (993 ± 449 for the DMPs and 451 ± 212 for the PMPs). For the DMP approach we additionally evaluated PI^2^ for policy search, which could not find as good solutions as the CMA policy search approach.

Next, we again want to assess the learning speed of both approaches. We again used CMA policy search for the PMPs and DMPs as well as PI^2^ for the DMP approach. The average over 20 runs of the learning curves are illustrated in Figure 9. Using the PMPs as movement representation, good policies could be found at least one order of magnitude faster compared to the trajectory-based DMP approach. The quality of the found policies was better for the PMP approach (mean values and standard deviations after learning: 993 ± 449 for the DMPs and 451 ± 212 for the PMPs). For the DMP approach we additionally evaluated PI^2^ for policy search, however, PI^2^ was not able to find good solutions—the robot always fell over.

#### 4.2.3. Generalizability to different task settings

In the next step we again test the generalization to different initial or final states. More specific we investigate how well the approaches can adapt to different priors of the arm joint.

**Figure 10:**
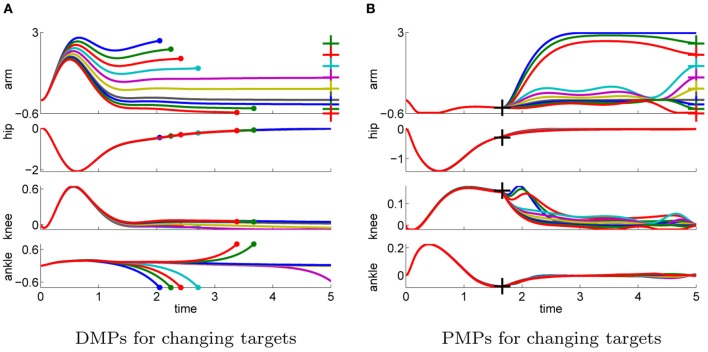
**This figure illustrates the joint angle trajectories (arm, hip, knee, and ankle) of a 4-link robot model during a balancing movement for different final targets of the arm joint (3, 2.5, 2, 1.5, 1, 0.5, 0, −0.2, −0.4, −0.6).** The applied policies were learned for a final arm posture of ϕ_*T*_arm__ = 0. **(A)** The valid range of the arm joint using DMPs is ϕ_*T*_arm__ ∈ [−0.2, 1]. Large dots in the plot indicates that the robot has fallen. **(B)** PMPs could generate valid policies for all final arm configurations.

In the previous task the target was assumed to be known prior knowledge and the policy was learned for a final arm posture of ϕ_*T*_arm__ = 0. We used this learned policy to generate movements to different final targets of the arm joint ϕ_*T*_arm__ ∈ {3, 2.5, 2, 1.5, 1, 0.5, 0, −0.2, −0.4, −0.6}. We only change either the arm-position of the last via-point or the point attractor of the dynamical system. The results shown in Figure 10 confirm the findings of the one-dimensional via-point task. The PMPs first move to the via-point, always maintaining the extracted task constraints, and afterward move the arm to the desired position while keeping balance. All desired target positions of the arm could be fulfilled. In contrast, the DMPs managed to keep balance only for few target positions. The valid range of the target arm position with DMPs was ϕ_*T*_arm__ ∈ [−0.2, 1]. This shows the advantage of generalization while keeping task constraints versus generalization per using the DMP heuristics.

**Figure 11:**
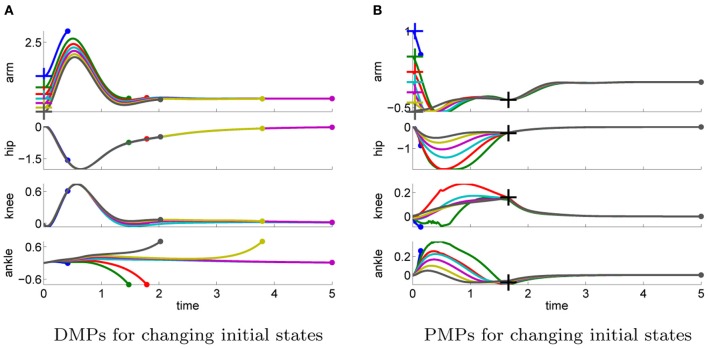
**This figure illustrates the joint angle trajectories (arm, hip, knee and ankle) of a 4-link robot model during a balancing movement for different initial states of the arm joint (1, 0.5, 0.2, 0, −0.2, −0.4, −0.6).** The applied policies were learned for an initial arm posture of ϕ_1_arm__ = 0. **(A)** The valid range of the arm joint using DMPs is ϕ_1_arm__ ∈ [−0.2, 0]. Large dots in the plot indicates that the robot has fallen. **(B)** PMPs could generate valid policies for ϕ_1_arm__ ∈ [−0.6, 0.5].

The ability of the two approaches to adapt to different initial states is illustrated in Figure 11. We used the learned policy for ϕ_1_arm__ = 0 to generate movements using different initial states of the arm joint: ϕ_1_arm__ = {1, 0.5, 0.2, 0, −0.2, −0.4, −0.6}. The push perturbing the robot results in an immediate change of the joint velocities, which are shown in Table A5 in the Appendix for these different initial states. For the DMPs only the joint angles 0 and −0.2 resulted in successful policies. Whereas with PMPs the valid range of the initial arm position was ϕ_1_arm__ ∈ [−0.6, 0.5].

#### 4.2.4. Model learning using PMPs

**Figure 12:**
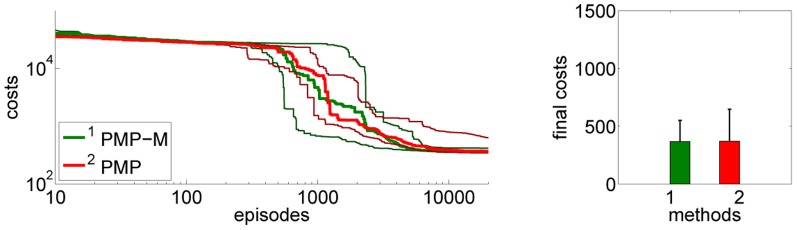
**This figure shows the influence of model learning on the 4-link balancing task.** Illustrated are mean values and standard deviations over 20 runs. The learning performance with the given system model is denoted by PMP. Instead of using the given model we now want to learn the system model from data (as described in section 3.3), which is denoted by PMP-M. In the beginning of learning the extrinsic costs are larger compared to motor skill learning with a given analytic model. However, as the number of collected data-points $\langle [x_{t};u_{t}],{\dot{x}}_{t}\rangle$ increases the PMPs with model learning quickly catch up and converge finally to the same costs. The PMP representation with model learning in parallel considerably outperforms the trajectory-based DMP approach in learning speed and in the final costs.

So far all experiments for the PMPs were performed using the known model of the system dynamics, these experiments are denoted by PMP in Figure 12. Note that also for the DMPs the known system model has been used for inverse dynamics control. Now we want to evaluate how model learning affects the performance of our approach. This can be seen in Figure 12. In the beginning of learning the extrinsic costs are larger compared to motor skill learning with a given analytic model. However, as the number of collected data-points $\langle [x_{t};u_{t}],{\dot{x}}_{t}\rangle$ increases the PMPs with model learning quickly catch up and converge finally to the same costs. The PMP representation with model learning in parallel considerably outperforms the trajectory-based DMP approach in learning speed and in the final costs.

#### 4.2.5. Computational time

For our simulations we used a standard personal computer (2.6 Ghz, 8 Gb ram) with implementations of the algorithms in C++. For the 4-link pendulum the DMPs could generate the movement trajectory within less than 0.1 s. With the proposed PMPs it took less than 1 s (including model learning). The time horizon was 5 s and we used a time-step of Δ*t* = 10 ms.

#### 4.2.6. Concluding summary

In contrast to the via-point task the optimal solution for this dynamic balancing task is unknown. The comparison to the DMPs shows a similar result as for the via-point task. With the PMPs we could find movements of significantly higher quality compared to the DMPs and the motor skill could be learned up to one order of magnitude faster with PMPs. We again applied the same parameters to different initial or final states to demonstrate the generalization ability. Now we can see the advantage of the learned task-relevant features. While the PMPs still try to fulfill the extracted task-relevant constraints and therefore succeeded for almost all initial/final state configurations, the DMPs again just heuristically scale the trajectory, which results in the robot falling in almost all but the learned configurations. Finally we showed that the dynamic model could be learned in parallel for the 4-link balancing task.

**Figure 13:**
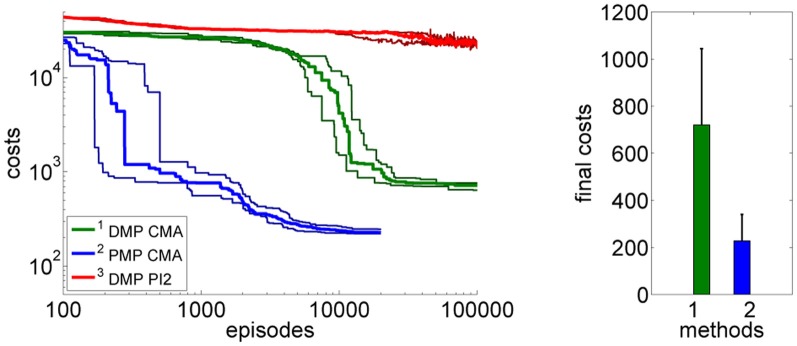
**This figure illustrates the learning performance of the two movement representations, DMPs and PMPs for the 4-link balancing task. But in contrast to the evaluations shown in the experimental section did we here apply a *negative* force F = −25 Ns.** Illustrated are mean values and standard deviations over 20 runs after CMA policy search. The controls (torques) are perturbed by zero-mean Gaussian noise with σ = 10 Nm. Using the proposed Planning Movement Primitives good policies could be found at least one order of magnitude faster compared to the trajectory-based DMP approach. Also, the quality of the best-found policy was considerably better for the PMP approach (720±323 for the DMPs and 227 ± 112 for the PMPs). For the DMP approach we additionally evaluated PI^2^ for policy search which could not find good policies.

**Figure 14:**
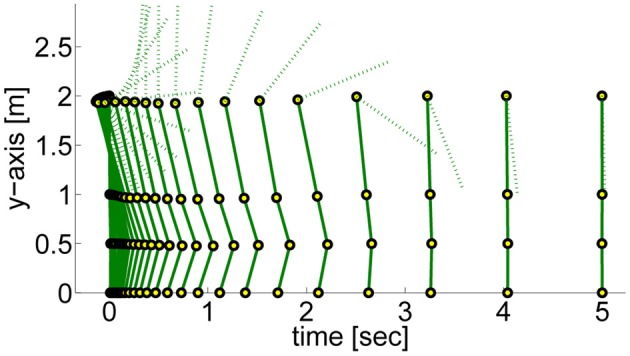
**This figure illustrates a dynamic balancing movement learned using the proposed Planning Movement Primitives.** The 4-link robot modelling a humanoid (70 kg, 2 m) gets pushed with the specific force (*F* = −25 Ns) and has to move such that it maintains balance. The circles denote the ankle, the knee, the hip, and the shoulder joint.

For all balancing experiments shown in this section the robot was pushed with the specific force *F* = 25 Ns. We have performed the same evaluations for various forces and the results are basically the same. For example, a comparison of the learning performances using the negative force *F* = −25 Ns is shown in Figure 13. The executed movement of the best learned policy using PMPs is shown in Figure 14.

In total 40 weights and 8 control gains were learned with DMPs. For PMPs we used 2 via-points where the second one was fixed. Thus, we learned 8 parameters specifying the first via-point and additionally 12 importance weights for each via-point. This results in 32 parameters.

## 5. Discussion

In this paper we concentrated on three aspects of biological motor control, which are also interesting for robotic motor skill learning: (1) the modularity of the motor system, which makes it possible to represent high-dimensional action spaces in terms of lower-dimensional MPs, (2) its variability and behavior under stochasticity, and (3) the efficiency in learning movement strategies.

In order to achieve similar properties also for robotic motor skill learning we propose to exploit the power of SOC already at the level of the MP. Instead of endowing a MP with a dynamical system, like the DMPs (Schaal et al., 2003), we endow a MP with an intrinsic probabilistic planning system. The resulting MP is called PMP. For the dynamical systems approach the parameters of the MP indirectly define the shape of the trajectory. In our case, the parameters of the MP define the intrinsic cost function of a graphical model, which represents a SOC problem. Performing inference in this graphical model yields the controls for executing the movement.

Due to the use of the intrinsic planning system our representation complies with basic principles of SOC. For example, the PMPs are able to account for the motor variability often observed in human motion. Instead of suppressing the noise of the system by following a single reference trajectory, the PMPs are able to learn to intervene the system only if it is necessary to fulfill a given task, also known as the minimum intervention principle (Todorov and Jordan, 2002). This allows a much higher variance in parts of the trajectory where less accuracy is needed. Current methods which rely on a reference trajectory are not able to reproduce these effects.

The parameters of PMPs encode learned task-relevant features of the movement, which are used to specify the intrinsic cost function for the MP's intrinsic planning system. Our experiments have shown that such a task-related parameterization facilitates learning and generalization of movement skills. Policies of higher quality could be found an order of magnitude faster than with the competing DMP approach. In addition, as confirmed by our experiments, the intrinsic planner also allows a wide generalization of the learned movement, such as generalization to different initial or goal positions. The DMPs on the other hand have to use heuristics for this generalization (Pastor et al., 2009), which had the consequence that the robot typically fell over in a new situation. In this case relearning is needed for the DMPs while the PMPs allow to reuse the learned parameters.

In traditional SOC methods (Todorov and Li, 2005; Kappen, 2007; Toussaint, 2009) the intrinsic cost function is typically hand-crafted. In contrast we learn the cost function from experience. We considered a general class of motor skill learning tasks where only a scalar reward is observed for the whole movement trajectory. Thus, with PMPs this external sparse reward signal is used to learn the intrinsic cost function. We applied the second order stochastic search method CMA (Hansen et al., 2003) for finding appropriate intrinsic cost functions. In this paper we focused on the representation of movements, and placed less emphasis on a specific policy search method. We want to point out again that our method does not depend on the used policy search method, any episode-based exploring policy search method can be used. We also do not want to argue for using episode-based exploring methods for policy search, however, as our experiments show, these methods provide useful alternatives to the more commonly used step-based approaches such as the PI^2^ algorithm (Theodorou et al., 2010). Future work will concentrate on more grounded approaches for extracting immediate costs from a sparse reward signal. This can also be of interest for imitation learning, where we do not know the immediate costs used by the demonstrator, but often we can evaluate the demonstrators behavior by an external reward signal.

The planner requires to know the system dynamics, which we also learn from data. As shown by our experiments this can be done without significant loss of performance. Hence, our approach combines model-based and model-free RL. As in model-based RL, we learn a system model to plan with. Model-free RL is used as method to search for appropriate intrinsic cost functions. We used the LWR (Atkeson et al., 1997) for learning the system dynamics as it is a very simple and effective approach. Future work will also concentrate on more complex robot models where more sophisticated methods like Vijayakumar et al. (2005), Nguyen-Tuong et al. (2008a), and Nguyen-Tuong et al. (2008b) could be applied for model learning.

In our experiments the number of phases was fixed (*N* = 2). It was assumed as prior knowledge and can model the complexity of the movement representation (Similarly, the complexity of DMPs can be scaled by the number of Gaussian activation functions). During our experiments we also evaluated the balancing task with up to *N* = 5 phases, but more than 2 phases did not improve the quality of the learned policy. One via-point on the other hand was not sufficient to describe the movement.

A promising topic for future investigation is the combination of primitives in order to achieve several tasks simultaneously. This is still a mostly unsolved problem for current movement representations. Because of the non-linear task demands and system dynamics a naive linear combination in trajectory space usually fails. Here, our PMPs offers new opportunities. New movements can be inferred by a linear combination of cost functions, which results in a non-linear combination of the policies for the single tasks.

## Conflict of interest statement

The authors declare that the research was conducted in the absence of any commercial or financial relationships that could be construed as a potential conflict of interest.

## Footnotes

^1^The bandwidth parameter for controls is set to zero as the matrizes **A** and **B** in the linearized model, i.e., $\dot{x}=A(x)x+B(x)u$ are independent on the controls **u**.

^2^The control gains, i.e., the two scalars *k*_pos_ and *k*_vel_ of the linear feedback controller in Equation (7) in the Appendix are learned using a 2nd order stochastic search method.

^3^For PMPs again 2 via-points were optimal. DMPs performed best when using 10 Gaussian kernels per dimension.

## Appendix

### A. Dynamic movement primitives

The most prominent representation for movement primitives (MPs) used in robot control are the DMP (Schaal et al., 2003). We therefore used the DMPs as a baseline in our evaluations and will briefly review this approach in order to clarify differences to our work. For our experiments we implemented an extension of the original DMPs (Pastor et al., 2009), which considers an additional term in the dynamical system which facilitates generalization to different target states. For more details we refer Schaal et al. (2003) to and Pastor et al., (2009).

DMPs generate multi-dimensional trajectories by the use of non-linear differential equations. The basic idea is to a use for each degree-of-freedom (DoF) of the robot a globally stable, linear dynamical system which is modulated by learnable non-linear functions *f*:

$$\tau \dot{z}=\alpha_{z}\beta_{z}(g-y)-\alpha_{z}z-\alpha_{z}\beta_{z}(g-y_{1})s+f,\tau \dot{y}=z,$$

where the desired final position of the joint is denoted by *g* and the initial position of the joint is denoted by *y*_1_. The variables *y* and $\dot{y}$ denote a desired joint position and joint velocity, which represent our movement plan. The temporal scaling factor is denoted by τ and α_*z*_ and β_*z*_ are time constants. The non-linear function *f* directly modulates the derivative of the internal state variable *z*. Thus, *f* modulates the desired acceleration of the movement plan. *s* denotes the phase of the movement.

For each DoF of the robot an individual dynamical system, and hence an individual function *f* is used. The function *f* only depends on the phase *s* of a movement, which represents time, $\tau \dot{s}=-\alpha_{s}s$. The phase variable *s* is initially set to 1 and will converge to 0 for a proper choice of τ and α_*s*_. With α_*s*_ we can modulate the desired movement speed. The function *f* is constructed of the weighted sum of *K* Gaussian basis functions Ψ_*i*_

$$f(s)=\frac{\sum_{i=1}^{K}\Psi_{i}(s)w_{i}s}{\sum_{i=1}^{K}\Psi_{i}(s)},\;\Psi_{i}(s)=\exp(-\frac{1}{2\sigma_{i}^{2}}{\left(s-c_{i}\right)}^{2}).$$

As the phase variable *s* converges to zero also the influence of *f* vanishes with increasing time. Hence, the dynamical system is globally stable with *g* as point attractor.

In our setting, only the linear weights *w*_*i*_ are parameters of the primitive which can modulate the shape of the movement. The centers *c*_*i*_ specify at which phase of the movement the basis function becomes active and are typically equally spaced in the range of *s* and not modified during learning. The bandwidth of the basic functions is given by σ^2^_*i*_.

Integrating the dynamical systems for each DoF results into a desired trajectory $\langle y_{t},{\dot{y}}_{t}\rangle$ of the joint angles. We will use an inverse dynamics controller to follow this trajectory (Peters et al., 2008). The inverse dynamics controller receives the desired accelerations ${\ddot{q}}_{\text{des}}$ as input and outputs the control torques **u**. In order to calculate the desired accelerations we use a simple decoupled linear PD-controller

(7) $${\ddot{q}}_{\text{des}}=\text{diag}(k_{\text{pos}})(y_{t}-q_{t})+\text{diag}(k_{\text{vel}})({\dot{y}}_{t}-{\dot{q}}_{t}).$$

Unfortunately standard inverse dynamics control did not work in our setup because we had to deal with control limits of multi-dimensional systems. Thus, we had to use an inverse dynamics controller which also incorporates control constraints. For this reason we performed an iterative gradient ascent using the difference between the actual (using constrained controls) and the desired accelerations ${\ddot{q}}_{\text{des}}$ as error function. This process was stopped after at most 25 iterations.

For our comparison, we will learn the linear weights **w** for each DoF as well as the controller gains **k**_pos_ and **k**_vel_, i.e., θ = [**w**_1_, …, **w**_*D*_, **k**_pos_, **k**_vel_]. This results into *KD* + 2*D* parameters for the movement representation, where *D* denotes the number of DoF of the robot.

### B. Task settings and parameters

In this section the MP parameters and constants are specified for the one-dimensional via-point task and for the humanoid balancing task.

#### B.1 One-dimensional via-point task

For the one-dimensional via-point task the parameters of the Dynamic Movement Primitives are listed in Table A1. The valid configuration space for the policy search algorithm is listed in Table A2. The CMA policy search algorithm has just one parameter, the exploration rate. Where the best exploration rate using DMPs for this task found was 0.05.

**Table A1:** **Via-point task: DMP movement primitive parameters**.

**K**	**α_*s*_**	**α_*z*_**	**β_*z*_**	τ
10	1	2	0.9	0.1

**Table A2:** **Via-point task: DMP policy search configuration parameters**.

	**w**	**k**_pos_	**k**_vel_
Lower bound	−100	0	0
Upper bound	+100	100	100

The limits of the parameterization of the Planning Movement Primitives (PMPs) (see Equation 4) is listed in Table A3. For the via-point task we choose *N* = 2, where the second via-point *g*^[*N*]^ = *g*_*T*_ was given. The exploration rate was set to 0.1 in all experiments.

**Table A3:** **Via-point task: PMP policy search configuration parameters with *i* = 1,2**.

	**d^[1]^**	**g^[1]^**	**r^[*i*]^**	**h^[*i*]^**
Lower bound	0.05	−2	[1, 10^−6^]	10^−4^
Upper bound	0.4	+2	[10^6^, 10^4^]	10^−2^

#### B.2 Dynamic humanoid balancing task

The DMP parameters for the balancing task are listed in Table A4. The policy search parameters are the same like for the via-point task, Table A2. The exploration rate was set to 0.1.

**Table A4:** **Balancing task: DMP movement primitive parameters**.

**K**	**α_*s*_**	**α_*z*_**	**β_*z*_**	τ
10	1	5	5	1

The PMPs were again evaluated with *N* = 2 via-points, where the second via-point *g*^[*N*]^ = *g*_*T*_ (the up-right robot posture) was given and for the first via-point the valid joint angle configuration is shown in Table 1 in Rückert and Neumann (2012). The exploration rate was 0.1 and the policy search algorithm configuration is listed in Table A5.

**Table A5:** **Balancing task: PMP policy search configuration parameters with *i* = 1, 2**.

	**d^[1]^**	**r^[*i*]^**	**h^[*i*]^**
Lower bound	0.1	10^−2^ for angles and 10^−4^ for velocities	10^−9^ **1**
Upper bound	4.6	10^4^ for angles and 10^2^ for velocities	10^−3^ **1**

Vector ***1*** denotes a 4-dimensional column vector, where all elements are equal to 1.

In the generalization experiment we applied the same learned policy of the 4-link balancing task to different initial states. For different initial arm joint configurations the push modulated with *f* resulted in different initial joint velocities, which are shown in Table A6.

**Table A6:** **Initial velocities (multiplied by 10^2^/*F*) for different initial states of the arm joint ϕ_0_arm__**.

**ϕ_0_arm__**	**Arm**	**Hip**	**Knee**	**Ankle**
1	−2.39	7.56	−8.54	1.42
0.5	−3.61	6.12	−7.9	1.32
0.2	−4.15	5.29	−7.52	1.25
0	−4.27	5.09	−7.43	1.24
−0.2	−4.15	5.29	−7.52	1.25
−0.4	−3.82	5.8	−7.75	1.29
−0.6	−3.43	6.38	−8	1.34
